# Ultrasonography Monitoring of Trauma-Induced Heterotopic Ossification: Guidance for Rehabilitation Procedures

**DOI:** 10.3389/fneur.2018.00771

**Published:** 2018-09-13

**Authors:** Qing Wang, Peizhen Zhang, Pengdong Li, Xiangfen Song, Huijing Hu, Xuan Li, Wufan Chen, Xiaoyun Wang

**Affiliations:** ^1^Department of Biomedical Engineering, Southern Medical University, Guangzhou, China; ^2^Guangdong Work Injury Rehabilitation Center, Guangzhou, China; ^3^Guangdong Provincial Key Laboratory of Medical Image Processing, Southern Medical University, Guangzhou, China; ^4^Nanfang Hospital, Southern Medical University, Guangzhou, China

**Keywords:** heterotopic ossification, ultrasonography, trauma, rehabilitation, diagnosis

## Abstract

Traumatic injury is one of varying causes of heterotopic ossification (HO). After HO occurrence, rehabilitation training need alterations to avoid the aggravation of HO. Therefore, monitoring of HO development plays an important role in the rehabilitation procedure. The aims of this study are to evaluate the post-traumatic HO occurring at various joints, to describe the features of HO development in ultrasound images, and to provide a guidance for the orthopedist to make individualized rehabilitation therapy. Eight subjects with the post-traumatic HO were recruited in this study. The joints on the injured side was examined by plain radiography. The joints on the injured side and the corresponding sites on the uninjured sides were scanned by ultrsonography. The HO tissues were segmented automatically using a semi-supervised segmentation algorithm. Then the HO tissues were evaluated in comparison with the corresponding region of the uninjured side. During the development stage of immature HO, ultrasonography was sensitive to observe the involved soft tissue and the calcification of HO. The characteristics of HO tissues in ultrasound image included the hyperechoic mass occasionally accompanied with acoustic shadow and the irregular muscular architecture. It was found that the mean grayscale value of HO was significantly higher (*p* < 0.001) than that of the uninjured side at the middle and late stages. During the development period of HO, the HO grayscale value gradually increased and the mean grayscale of value of mature HO was significantly higher (*p* < 0.05) than that of immature HO. According to the information of HO provided by ultrasound, the orthopedist properly adjusted the rehabilitation treatment. The results demonstrated that the visualization of HO using ultrasonography revealed the development of HO in the muscle tissues around the injured joints and thus provide a guidance for the orthopedist to make individualized rehabilitation therapy. Ultrasound could be a useful imaging modality for quantitative evaluation of HO during the rehabilitation of traumatic injury.

## Introduction

Heterotopic ossification (HO) has been clinically described as lamellar bone formation in the periarticular soft tissues, where osseous tissue should not exist. This aberrant bone formation is commonly associated with orthopedic interventions, trauma, stroke, traumatic brain injury, spinal cord injury, and neurological disorder. Therefore, it is summarized under three ways of acquiring HO: traumatic, non-traumatic (very rare), or neurogenic. However, the definitive pathogenesis of HO is still quite unclear. Early HO causes restriction of joint movement, local pain and swelling. The clinical symptoms of early superficial HO may include erythema and localized warmth (1, 2). At the late stage of HO, the mature osseous tissues lead to severe limitation of the range of motion of the joint, pain in the affected joints, and even nerve or vessel compressed by HO (2).

In the clinical laboratory, biochemical markers such as serum alkaline phosphatase (AP) and bone alkaline phosphatase (BAP) are usually examined to reveal the alterations in bone metabolism. The changes in biochemical markers reflect the bone formation or bone resorption, but could not provide visualization of the change of bone (3). Citak et al. proved that laboratory findings of elevated AP and BAP might not be reliable for early HO detection because of their low diagnostic specificity (4). Therefore, the laboratory examinations are not suggested to be routine examination.

Nowadays, several imaging modalities have been applied to evaluate HO. In department of orthopedics in a hospital, plain radiography is usually used to locate and visualize the heterotopic bone tissues. Plain radiograph is considered as the gold standard of the clinical diagnosis of HO because it is inexpensive and convenient to detect HO. Computed tomography (CT) is sometimes used to provide 3-D information of HO and clearly observe the location and volume of HO. However, the cost of CT exam is high. Patients are exposed to a higher dose of radiation by plain radiography and CT, which only provide the diagnosis of HO in the late stage that the osseous tissues develop into the matured bone. Therefore, these two imaging modalities are unable to detect ossification in the early inflammatory stage. The HO progression needs to be further evaluated (2). Magnetic resonance imaging (MRI) is useful for imaging soft tissue in the early stage of HO. Ledermann et al. proposed a HO grading scale based on MRI characteristics to evaluate the development of HO: grade 1 = fluid attenuation without calcifications, grade 2 = calcification of soft tissue without evidence of bone formation, grade 3 = immature bone formation, and grade 4 = mature bone with cortical formation (5). However, MRI has low resolution and is expensive and relatively insensitive to the bone tissues.

The advantages of ultrasonography are that ultrasound avoids ionizing radiation, and is widely available, repeatable and inexpensive for bedside monitoring (6). It is proved that ultrasound is a sensitive imaging method in evaluation of soft tissue lesions and calcifications (7). Some previous studies utilized ultrasonography to evaluate the muscle tissues by measurements of grayscale value and thickness of the muscles (8, 9). Furthermore, the relationship between ultrasound measurements and the diseases were explored (8, 10). A recent study revealed the negative correlation between the echo intensity of the rectus femoris and muscle strength (11). Other previous studies demonstrated that ultrasound could be effective for detecting immature HO. Bedside ultrasound were applied to diagnose immature HO caused by brain or spinal cord injury and the results suggested that ultrasonography could be a useful first-line imaging modality in the diagnosis of early HO (12–14). Recent studies stated that ultrasonography could distinguish matured HO from the surrounding soft tissues with a high specificity (15–17). Trauma leading to artificial joint replacement and internal fixation of bone fracture without neurological injury, may also cause HO. The rehabilitation outcome of the injured joints may be greatly affected by HO. Therefore, ultrasonography could be applied in orthopedic rehabilitation for providing a guidance for the orthopedist to make individualized rehabilitation therapy. However, quantitative evaluation of HO and serial follow-up ultrasonography for depicting HO progression were rarely reported.

In this study, ultrasonography was applied to observe the calcification in soft tissues in the participants with the post-traumatic HO caused by trauma such as bone fracture and contusion in the rehabilitation center. The aims of this study are to evaluate the post-traumatic HO occurring at various joints, to describe the features of HO development in ultrasound images, and to provide a guidance for the orthopedist to make individualized rehabilitation therapy. The ultrasonographic results were compared with those of plain radiography, which is the gold standard of the clinical diagnosis of HO in orthopedics.

## Materials and methods

### Participants

The participants were selected from Guangdong Work Injury Rehabilitation Center, China. The criteria of participants include the following: (I) trauma such as fractures, dislocations and contusion occurring at the joints, (II) ossification in soft tissues surrounding the injured joint but not attaching to the cortical bone visualized by radiograph, and (III) motion restriction and pain of the affected joints. Eight participants (6 males and 2 females, Age: 23–48) with symptoms of HO were recruited. The exclusion criteria include the following: (I) inadequate acquisition of ultrasound images, and (II) osteochondroma. The basic information for the participants is shown in Table 1. Four participants had post-traumatic HO at the elbow joint (50%), 3 at knee joint (37.5%), and 1 at shoulder joint (12.5%).

**Table 1:** Background data of the participants.

**Participant No**.	**Age (years)**	**Injured joint**	**Traumatic injury**	**Range of joint motion**
1	30–35	Right elbow	Bone fracture of distal humerus & head of radius	Extension: 50°; flexion: 85°
2	20–25	Left shoulder	Bone fracture of humerus surgical neck	Limited motion range of left shoulder joint
3	40–45	Left knee	Splintered bone fracture of patella with collateral & cruciate ligament injury	Extension: 5°; flexion: 15°
4	44–49	Left elbow	Bone fracture of lateral epicondyle of humerus with muscle strength reduction	Extension: 25°; flexion: 110°
5	45–50	Right knee	Soft tissue avulsion & contusion of proximal end	Flexion: 25°
6	43–48	Right elbow	Splintered bone fracture of distal humerus	Extension:30°; flexion: 40°; adduction: 70°; abduction: 50°
7	40–45	Right elbow	Splintered bone fracture of ulnar coronoid process & head of radius	Extension: 30°; flexion:105°; adduction: 10°; abduction: 40°
8	45–50	Left knee	Bone fracture of tibial plateau	Extension: 10°; flexion: 55°

The clinical assessments of the range of joint motion were conducted by an experienced physiotherapist. The study was approved by the Ethics Committee of the Guangdong Work Injury Rehabilitation Center, Guangzhou, China. All subjects provided written informed consent prior to enrollment.

### Apparatus

In this study, four participants No. 1, 2, 3, and 4 were scanned by a portable ultrasound system (Mindray M5, Mindray, Shenzhen, China) with a linear transducer (Mindray 7L4s, a range of central frequency from 5 to 10 MHz). The other four participants No. 5, 6, 7, and 8 were scanned by a wireless ultrasound probe with a fixed central frequency of 10 MHz (Uprobe-3N, linear transducer, SonoStar, Guangzhou, China). A pilot test was conducted to ensure that the B-mode grayscale images obtained by the two ultrasound systems were of similar image quality.

All participants underwent examination of plain radiography (Aristos VX plus, SIEMENS, Germany). Two participants No. 4 and 5 were also examined by CT to obtain more information of the injured joint to distinguish HO from osteochondroma.

### Procedure of ultrasound examination

**Figure 1:**
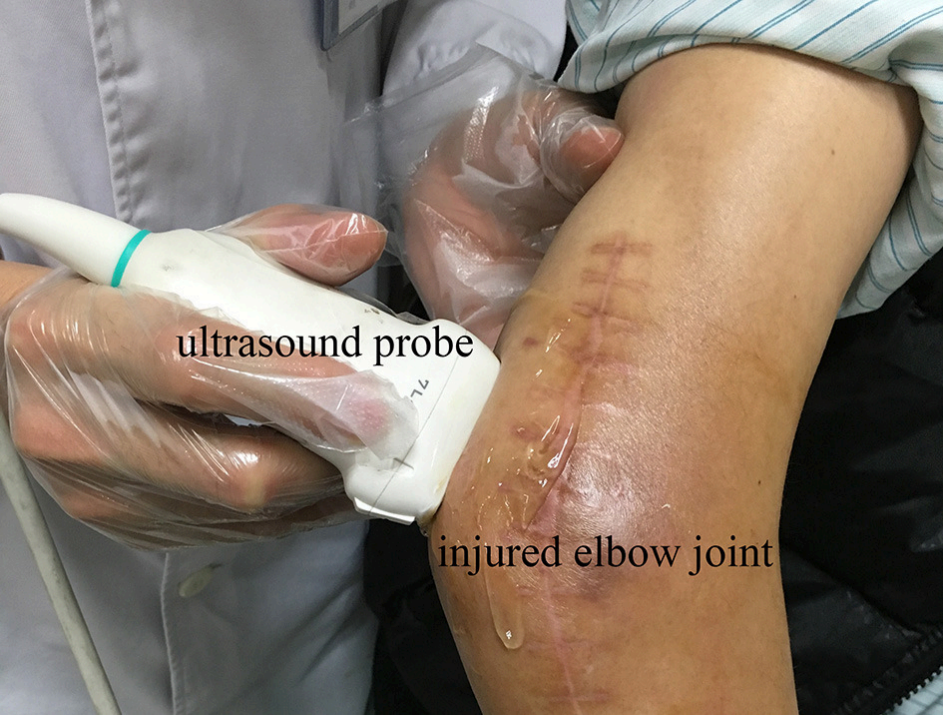
Longitudinal ultrasound scanning of HO at the posteriorlateral site of the injured elbow joint of participant No. 1.

After examination of plain radiography, all participants were examined by ultrasonography. First, the injured joint was scanned at the site of HO occurrence (Figure 1). The ultrasound probe was placed on the scan site with enough coupling gel interposed between the transducer and the skin. The depth of view of the ultrasonographic scan was set at 4 cm for the joints to clearly display the HO lesions and the structure of the muscles and bone. The gain was fixed at a constant intensity for all scans. All the ultrasound images were obtained respectively at the short and long axis of the muscles. Next, the same scanning procedure was performed at the corresponding site on the uninjured side as control.

Because participant No. 8 was with HO on the immature bone formation stage, he underwent the first ultrasound scan 2 days after the first plain radiography and the follow-up ultrasound scan was performed every week. Four follow-up scans were performed to monitor the development of HO. At each time point, five longitudinal and transverse ultrasound images of HO were obtained at the adjacent scan sites with an interval of approximately 0.5 cm, respectively. During this period of ultrasound monitoring, the participant was also underwent rehabilitation therapy. At the end of this study, the participant underwent an additional X-ray examination again.

For the participants with mature HO, the ultrasound scan was performed 2 days after plain radiography without the follow-up ultrasound scan.

### Segmentation and assessment of HO

**Figure 2:**
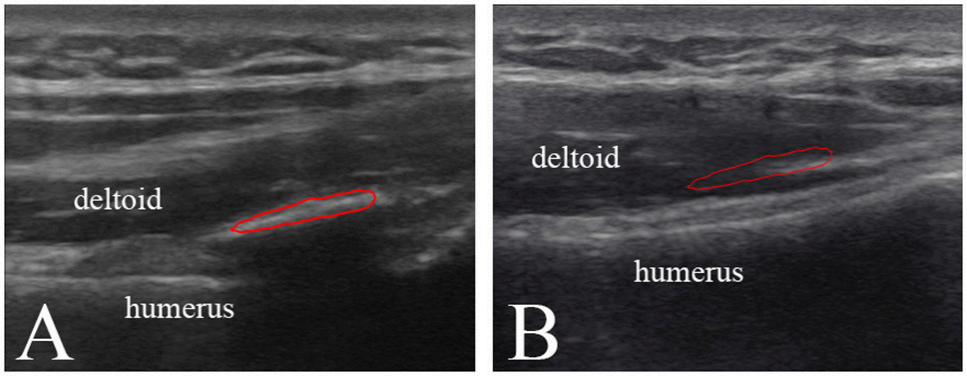
Ultrasound greyscale images of the injured left shoulder joint **(A)** and the uninjured right shoulder joint **(B)** of participant No. 2. Red profile in **(A)** and **(B)** represents the HO segmented from the surrounding soft tissues and the normal muscle tissue selected on the corresponding position, respectively.

Because the edge of the target tissue was not easily distinguished from the background in the ultrasound image, manual segmentation of the HO tissue was time consuming and operator dependent. Therefore, this study applied a semi-supervised segmentation algorithm based on patch representation and continuous min cut (18) to semi-automatically segment the region of interest (ROI) of HO in ultrasound image. Under semi-supervision of the clinical expertise, the HO can be accurately and specifically segmented from the surrounding soft tissues according to the texture features of the HO and muscles. Figure 2A shows the HO ROI segmented from the background in ultrasound grayscale images of the injured joint. Meanwhile, a same size ROI of the health tissues was selected at the corresponding position on the uninjured side (Figure 2B).

After segmentation of the tissues, the ultrasound characteristics of the images were assessed. The mean grayscale values of the HO tissue was quantitatively evaluated in comparison with the normal muscle tissue.

The segmentation and calculation of mean grayscale value were performed by a self-developed program using Matrix Laboratory (Matlab, version 2016b).

### Statistical test

The results of the pilot study showed that there was no significant difference between the grayscale values of the HO tissues in ultrasound images recorded using the two ultrasound systems. Therefore, the effect of ultrasound system on the measured grayscale values was not considered in this study.

The grayscale values were expressed as means and standard deviations (mean ± SD). Due to the small number of the participants, the non-parametric statistical tests were utilized in this study. The Mann–Whitney *U*-test was performed to test the statistical significance in the grayscale values between the HO and health muscle, and Friedman test was performed to test the statistical significance in the grayscale values measured on different time spots during the HO development period. The significance level was set at 0.05. This statistical analysis was performed by using the Statistical Package of Social Sciences (SPSS, version22, USA).

## Results

The results of plain radiography showed that seven participants were diagnosed with the post-traumatic mature HO and participant No. 8 was with the immature bone formation. Therefore, for participant No. 8, to track the alterations of the immature bone tissue during the development of HO, the follow-up ultrasound scans and plain radiography were performed.

### HO in the immature bone formation stage

**Figure 3:**
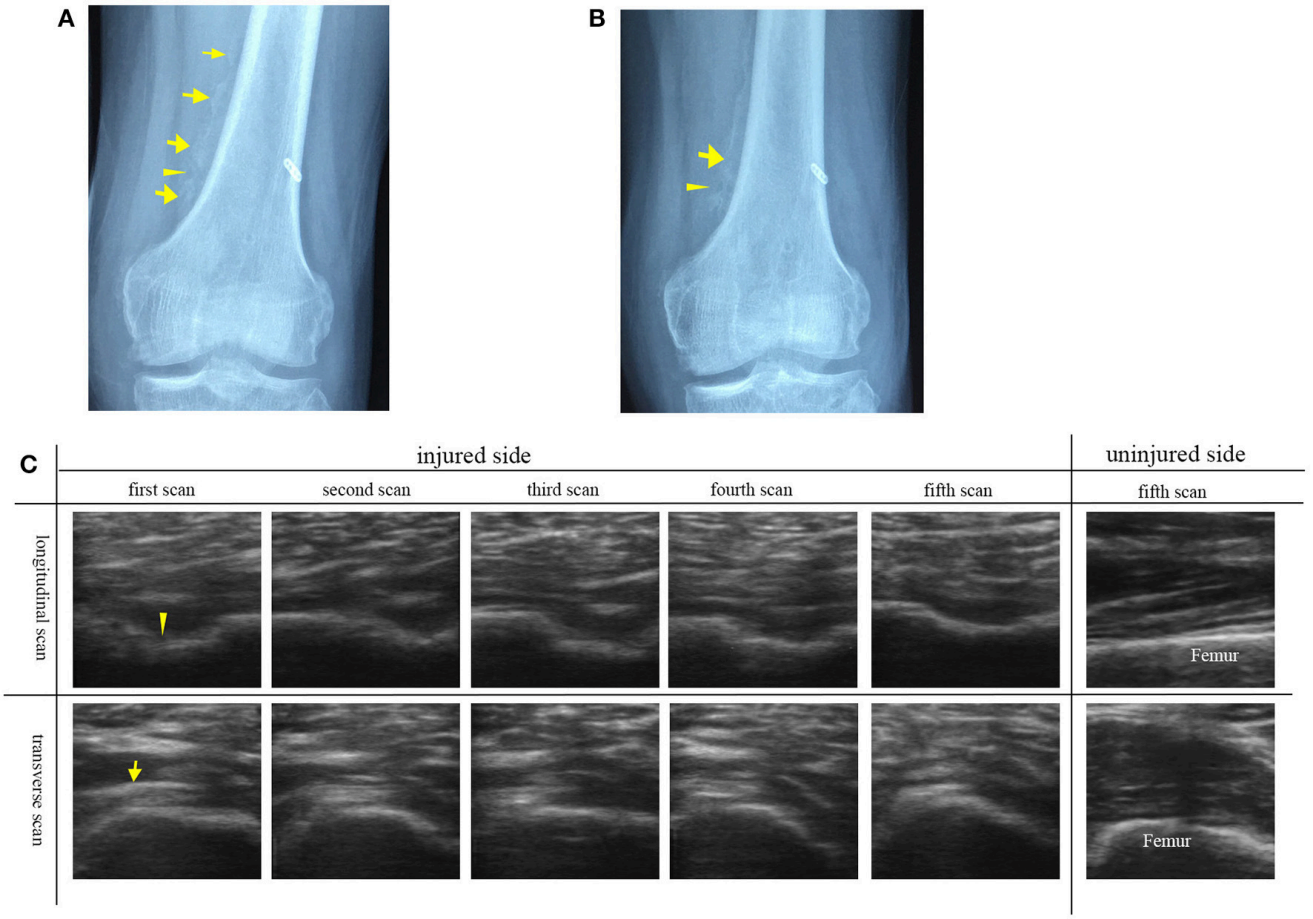
Plain radiographs and ultrasound images of left knee joint of participant No. 8 with HO in the immature bone formation stage. **(A)** The plain radiograph was taken as the participant was in hospital. Discontinuous (arrowhead), faint and poorly demarcated (arrows) calcification was found. **(B)** The second plain radiograph was taken ~1 month after the first plain radiograph. Continuous (arrowhead) and distinguishable (arrow) HO was found. **(C)** A series of transverse and longitudinal ultrasound images of the injured joint in comparison with the uninjured side. HO is visualized during its development. The unconnected HO tissues (arrowhead) correspond to the gap between two calcified HO tissues. On the fifth ultrasound images, the HO tissues connect smoothly to develop into the mature HO tissues. As the echogenicity and homogenesis of HO increased, the profile of the femur become blurred (arrow).

The participant No. 8 was in hospital because he suffered swelling and limited range of motion of left knee joint 4 months after surgery for trauma. Figure 3A shows discontinuous, faint, and poorly demarcated calcification indicating the immaturity of HO. Approximately 1 month after the first plain radiograph, the second plain radiograph showed that the HO tissues became continuous and distinguishable indicating the more ossified HO tissues, but still not mature enough for HO excision (Figure 3B).

In comparison with the plain radiographs, ultrasound images clearly showed the alterations in the anatomic, morphological structure of the involved soft tissues and HO tissues during the rehabilitation procedure (Figure 3C). From the transverse ultrasound images, mixed hyperechoic and hypoechoic areas in the swelling affected muscle tissues and loss of the texture pattern of muscular fibers in the early stage of immature bone formation compared with the health muscle tissues on the uninjured joint. During the development of HO, it was found that the echogenicity and homogenesis of the HO tissues increased as the ossification increased, and an acoustic shadowing caused by the HO tissues reduced the smoothness of the profile of the femur. Similarily, the longitudinal ultrasound images clearly visualized the development of the immature HO. During rehabilitation procedure, the discontinuous hyperechoic calcified tissues grew to become continuous lamellar HO tissue (Figure 3C). It was noted that the HO tissues in ultrasound image (Figure 3C) was consistent with lamellar bone in plain radiograph (Figures 3A,B). Ultrasonography is more sensitive to visualize the involved soft tissue and immature bone formation.

### HO in the mature bone formation stage

**Figure 4:**
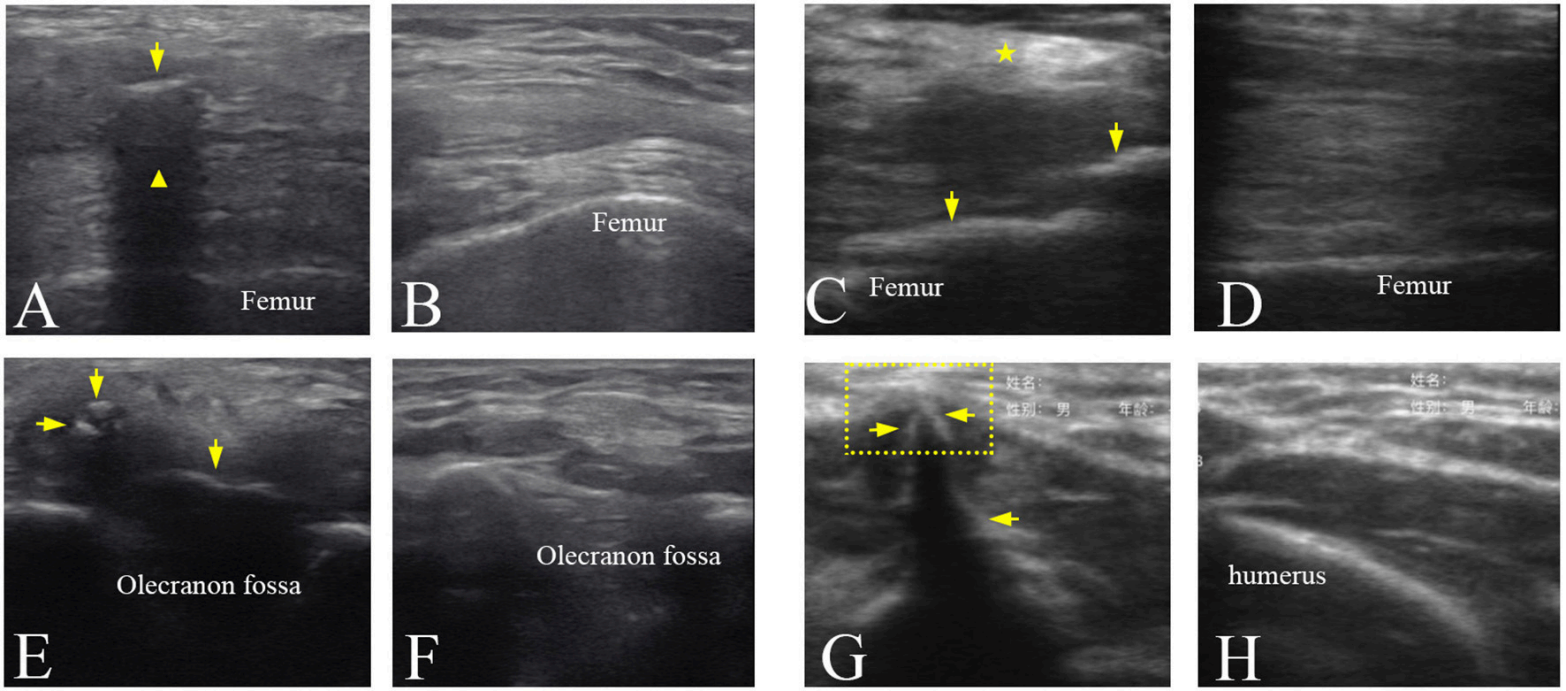
Ultrasound images of the mature HO in the different joints. **(A)** Ultrasound image of the left knee joint of participant No. 3 showing that HO (arrow) with an acoustic shadowing (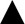). **(C)** Ultrasound image of the right knee joint of participant No. 5 showing a hyperechoic area (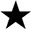) in the muscles and the HO tissues (arrows). **(E)** Ultrasound image of the right elbow joint of participant No. 4 showing the hypoechoic HO tissues (arrows) and loss of the textural structure of muscular fibers. **(G)** Ultrasound image of the right elbow joint of participant No. 6 showing local muscle evagination (dotted line box) by the hyperechoic HO tissues (arrows). **(B,D,F,H)** Ultrasound images of corresponding position of the uninjured side in **(A,C,E,G)**, respectively.

The ultrasound images of the injured joints show the irregular muscular architecture and the calcified foci (HO) (Figure 4). As Figure 4C shown, irregular hyperechoic areas and loss of deep fascia or aponeruroses in the swelling affected muscles were found in three participants No. 1, 5, and 8. Besides HO tissues, loss of the textural structure of the muscular fibers in the swelling affected muscles was found in participant No. 4 (Figure 4E). Figure 4G shows local muscle evagination due to the HO tissues in the muscles disturbing the muscle structure and causing worse pain in participant No. 6. With less injured muscles in two participates No. 2 and 3, the effect of the mature HO tissues on the texture of muscular fibers sometimes was not obvious (Figure 4A). The characteristics of mature HO in ultrasound image might include small calcified foci in size but with high echogenicity and sometimes with acoustic shadow. Figures 4B,D,F,H show the normal textural structure and echogenicity of the muscles and bones.

### Analysis of grayscale value

**Figure 5:**
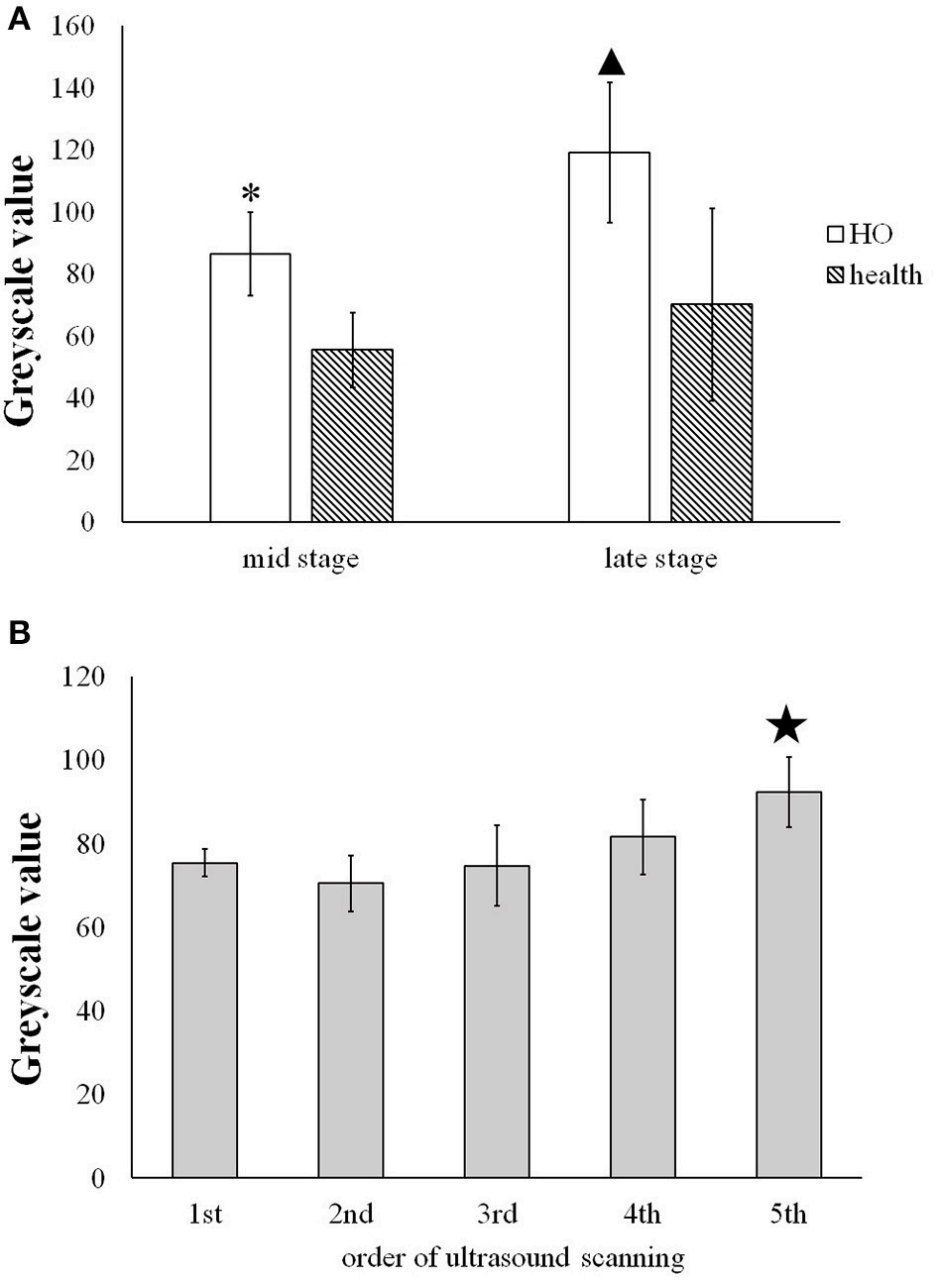
**(A)** Comparison of the greyscale value of the HO tissues and the health muscle tissues at the middle and late stage. * and 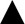 present the statistically significant difference between HO and health muscle (*p* < 0.001). **(B)** Comparison of the grayscale value of HO measured on five time spots during the development of HO. 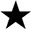 indicates statistically significant increase in the grayscale value of the 5th scan v.s. the former four scans (*p* < 0.05).

Figure 5A shows that at the middle stage of the in immature bone formation the grayscale value (86.40 ± 13.42) of the immature HO already increased significantly (*p* < 0.001) compared with the health muscle tissue (55.1 ± 12.01). At the late stage of HO, it was found that the grayscale value of the mature HO increased greatly (119.09 ± 22.70) and was significantly higher (*p* < 0.001) than that of to the health soft tissues (70.06 ± 31.09).

Figure 5B shows that the gradual increase in the grayscale value of the immature HO tissues during the development period. The grayscale value of the mature HO for 5th ultrasound scan (92.18 ± 8.37) was significantly increased in comparison with the 1st, 2nd, 3rd, and 4th scans (*p* < 0.05).

### Effect of HO on rehabilitation therapy

For the patients with high HO maturity, the strength of the rehabilitation therapy needs to be strengthened appropriately, in order to improve the joint movement function. However, for the patients with low HO maturity, the strength of rehabilitation treatments such as joint loosening and drafting should be soft in order to avoid locally strained muscles and other soft tissues, which might lead to HO worsened.

According to the ultrasound images, the orthopedist obtained the information of HO development of participant No.8. The individualized rehabilitation therapy was performed. The treatment intensity of the traditional rehabilitation training reduced. The participant was anesthetized and underwent with manipulation and arthroscopic surgery. After ~1-month rehabilitation therapy, the range of extension of the injured knee joint was 5–10, while the range of flexion increased 40° reaching up to 95°. The muscle strength also slightly increased from grade 4 to grade 4+. Furthermore, with the increase in maturity of HO, no obvious increase of HO size was observed via ultrasonography and plain radiography examinations.

## Discussion

### HO muscle and its neuromuscular function

Although the exact mechanism of HO in traumatic and neurogenic HO is unknown, two common factors precede the formation of HO (19). One factor is trauma or neurological injury. The other is the tissue expression of bone morphogenetic proteins (BMPs), which induce bone formation. In this study, all the participants sustained bone fracture at different joints with HO. We found that the morphologic and textural pattern of HO muscle changed in comparison with normal muscle. Differently, a recent study on spastic muscle induced by spinal cord injury did not find obvious changes in textural pattern of the involved muscle but only shortening of muscle fibers in ultrasound image (20). This may suggest that ultrasonography visualizing the damage in the textural pattern of the involved muscle is able to diagnose HO tissue.

Further, previous study reported that HO formation was related to a series of changes within not only muscles but also nerves and vessels (21). Those alterations may affect the neuromuscular function. After traumatic injury, the sensory nerves detect any damage to the bone, for example, the alterations due to trauma and BMPs. Then, the sensory nerves signal to the central nervous system to start the remodeling program. However, new bone is generated in incorrect places, and HO occurs. Therefore, the regulation of peripheral nerves system is involved in the formation of HO (21, 22).

### Quantitative assessment of grayscale value

Ultrasound images show characteristics, such as size and morphological structure, of the HO tissues. However, these characteristics are individually different in the different joints of different patients. Ultrasound echoes reflect the acoustic impedance, density, stiffness of the tissues. The magnitudes of the echoes are displayed in ultrasound image by grayscale values. During occurrence of HO, primitive mesenchymal cells transform into osteoblasts in the late stage. Previous studies demonstrated that the alterations in the properties of the tissues led to the changes in the ultrasound echoes reflecting the relative diseases (8–11). Therefore, this study chose grayscale value as quantitative parameter, which is relative to the maturity degree of the HO tissues (23). The results show the significant difference in grayscale values not only between the HO tissue and the health muscle tissue but also between immature and mature HO tissues.

### Radiographic classification and evaluation for HO

Previous studies evaluated and classified HO according to the plain radiograph. Based on radiographic findings, HO in the hip joint is grouped into four classes (Class I to Class IV), called as the Brooker classification (24). The Hastings and Graham classification system (25) was proposed to evaluate HO at the elbow joint into three classes. However, these classification systems were only applied to individual joint and did not obtain good classification results of HO in other anatomical locations.

A recent study proposed an analog scoring method (26) for radiographic classification and evaluation of HO based on normotopic reference bone. Its results showed high correlation (*R*^2^ = 0.89) between the scores of the analog scale and the heterotopic bone volumes measured by micro-CT, i.e., higher score means larger size of HO. In comparison with the analog method, this study evaluated grayscale value of HO in ultrasound image, indicating that higher echogenicity means more mature HO.

### Limitation of plain radiography

**Figure 6:**
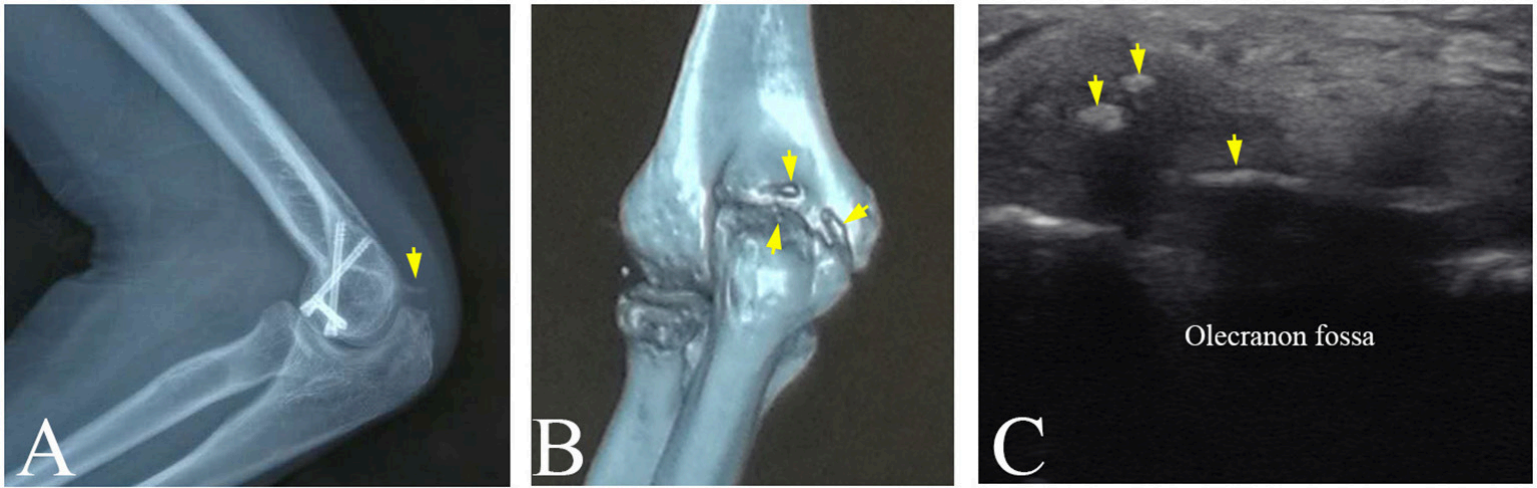
The HO tissues at left elbow joint of participant No. 4 visualized by multi-modality imaging. **(A)** A spiny HO (arrow) in plain radiograph. **(B)** A slender HO and two small and granular HOs discovered in CT image (arrows). **(C)** Two small hyperechoic masses and a slender hyperechoic region (arrows) in triceps brachii in ultrasound image.

In department of orthopedics in a hospital, plain radiography is considered as the gold standard of the clinical diagnosis of HO because of its convenience to detect the calcified tissues. However, plain radiography is a method of projection imaging producing two-dimensional images with x-ray radiation through the body tissues. Therefore, the plain radiograph does not provide any depth information. This limitation leads to misjudgment on HO. In this study, participant No.4 suffered from pain and limited range of motion in the left elbow joint. The radiography revealed a lamellated and slender calcification that intersected the distal humerus (Figure 6A). However, 3-D CT image visualized a slender HO and two small and granular HO tissues (Figure 6B). Similarily, ultrasonography also displayed two small hyperechoic calcified foci and a lameller hyperechoic region in triceps brachii (Figure 6C). It was found that the overlap of the HO tissues and distal humerus or other tissues in the radiograph might cause the misdiagnosis of the HO. Other previous studies slimilarily demonstrated that 3-D CT image help plain radiograph accurately locate the position and number of the HO tissues (27, 28).

### Advantages and limitations of ultrasonography in detection of HO

Previous studies have proven that ultrasound is a useful tool and provides an earlier diagnosis of neurogenic HO than radiography (12–14). This study similarily showed that ultrasonography could depict the changes of related soft tissues and immature HO tissues, that plain radiograph might fail to imagine in orthopedic rehabilitation. The hypoechoic area and loss of the lamellar pattern of muscular fibers in ultrasound image (Figure 4E) probably due to the inflammatory edema surrounding HO. Hyperechoic holistic muscle, especially hyperechoic muscular fibers (Figure 4C) might be caused by the lack of exercise or amyotrophy.

In addition, ultrasonography could be conveniently performed a bedside monitoring using portable or wireless ultrasound device. There was no significant difference between two devices for imaging and quantitatively evaluation of HO. This study demonstrates that grayscale value analysis method for evaluating HO is independent on ultrasound system. Finally, ultrasonography is suitable for follow-up tracking the development of HO due to its real-time imaging and no radiation.

However, operator dependency limits the use of ultrasonography (29), especially in department of orthopedics. Most orthopedists themselves have no experiences of performing ultrasound scanning and diagnosing HO using ultrasound image. And orthopedists are lack of the cooperation with ultrasound specialist. Another limitation of this study is the number of the proper participants. No participant with early stage of post-traumatic HO was included in this study, only one participant with the immature HO in the middle stage was involved.

## Conclusion

In this study, ultrasonography was applied to visualize the development of immature HO and surrounding soft tissues. After the HO extracted from the ultrasound images, the grayscale value was used to quantitatively assess the immature and mature HO tissues in the middle and late stage. The results show the significant difference in grayscale values not only between the HO tissue and the health muscle tissue but also between immature and mature HO tissues. This study suggested that ultrasonography has potentials to be a useful imaging modality for monitoring the development of HO and providing quantitative evaluation on HO. Combination of ultrasonography and plain radiography in diagnosis of HO help orthopedists to make individualized rehabilitation therapy.

## Author contributions

QW and XW conceived and designed the study. PZ and PL performed the experiments. QW and PZ wrote the paper. XS, XL, and HH contributed to experiments. XW and WC reviewed and edited the manuscript. All authors had read and approved the manuscript.

### Conflict of interest statement

The authors declare that the research was conducted in the absence of any commercial or financial relationships that could be construed as a potential conflict of interest.
